# How Miners and Other Professional Groups Perceive the Benefits and Risks of Hard Coal Mining: A Study on the Role of the Affect Heuristic

**DOI:** 10.3389/fpsyg.2021.656960

**Published:** 2021-06-29

**Authors:** Piotr Zielonka, Wojciech Białaszek, Bartłomiej Dzik, Katarzyna Wybrańczyk

**Affiliations:** ^1^Institute of Biology, Warsaw University of Life Sciences SGGW, Warsaw, Poland; ^2^DecisionLab, Institute of Psychology, SWPS University of Social Sciences and Humanities, Warsaw, Poland; ^3^Department of Economic Psychology, Kozminski University, Warsaw, Poland

**Keywords:** hard coal mining, environmental threats, affect heuristic, cognitive dissonance, energy sector, green shift, risk perception

## Abstract

The problems that are inherent in the green shift of the energy sectors are particularly visible in countries where the hard coal mining industry plays an important role in the economy and society. For any transition to be successful, public support is crucial. This empirical study shows that – as a consequence of the affect heuristic – those who perceive hard coal mining as beneficial tend to minimize both its detrimental environmental impacts and its personal safety hazards. Ignoring the affect heuristic may have retarded transformations and led to a failure of many information campaigns.

## Introduction

In modern democracies, the social acceptance of costly and ambitious environmental policies is crucial. There are social groups that object to the transformation from hard coal usage to green energy. They seem to perceive hard coal mining as beneficial. With this in mind, we examined their opinions not only on the benefits but also on the riskiness of hard coal mining.

The aim of the present study was to answer this question:

How do people involved in the hard coal mining industry perceive the economic benefits of hard coal usage, the environmental impact of hard coal mining, and the personal safety hazard of working underground?

People rely on feelings of “goodness” or “badness” in making judgments or decisions (Seabright, 2010). The judgment is initially quick, intuitive, and affective, which has a profound effect on our assessment of a decision problem. Rather than carefully calculating expected utilities, as the normative decision theory predicts, people perform a process which Finucane et al. (2000) call “rapid affective algebra.” The use of positive and negative impressions (affective tags) as cues in making judgments is known as the halo effect (Slovic et al., 2002). The fact that some decision processes are instant and not deliberative implies a shortcut of some sort; the judgment will likely be simplified, dominated by the most salient – at that moment – affective cue. This may be especially relevant in situations when different objects are judged separately rather than compared side by side – as demonstrated by Hsee (1998), who asked subjects how much they were willing to pay for a used music dictionary. Two exemplary dictionaries were used: Dictionary A with 10,000 entries in a perfect condition and Dictionary B with 20,000 entries but with a torn cover. When judging separately, Dictionary A was priced to be higher than Dictionary B – the torn cover elicited a negative affective impression and strongly devalued the bigger dictionary. However, when subjects judged the dictionaries side by side, they priced Dictionary B higher than Dictionary A because it was vastly superior in the most practically important dimension, the number of entries.

Decision theory assumes benefits and risks, which are appeared to be distinct concepts. In reality, benefits are usually positively correlated with risks. However, a common perception of the correlation is biased. If people exhibit positive feelings toward an activity or object, they are more likely to judge the benefits as high and the risk as low. On the other hand, if people have negative feelings toward an object, they are more likely to perceive the benefits as low and the risk as high. This bias is called the affect heuristic. The financial market provides a typical example whereby individual investors mistakenly perceive high expected returns as having a strong low-risk correlation (Shefrin, 2005).

The first empirical study by Finucane et al. (2000) showed that the affect suppresses the weighing of costs and benefits and introduces a unidirectional (entirely positive or negative) evaluation of the attributes of the object being assessed. Participants were asked to judge the benefits and risks of 23 different activities. A negative correlation between judgments of the benefit and risk was reported (the larger the benefit, the smaller the risk, and vice versa). Under time pressure, correlations were stronger as people who have less time to undertake an evaluation rely more on initial impressions rather than deliberation. The second study by Finucane et al. (2000) involved the manipulation of affective judgment by providing selective information about the risks or benefits of three different technologies: nuclear power, natural gas, and food preservatives. The authors speculated that providing information regarding only one attribute (benefit or risk) would form the evaluation of the other (non-manipulated) attribute in the same direction. Participants were provided with information, which aimed to increase or decrease the risk or benefit (four experimental conditions) of a technology. They were subsequently asked to perform their own evaluation. A negative correlation between the benefit and risk was obtained as a result. For example, if the information was created to decrease the risk of a given technology, participants perceived its benefit as being larger. On the other hand, if the information was created to decrease the benefit of a given technology, participants perceived its risk as being larger. The affect heuristic falls within the category of automatic information processing in terms of dual-processing theories, i.e., the fast and simplifying heuristics (Kahneman, 2011).

Siegrist and Sütterlin (2014) introduced a useful distinction regarding the affect heuristic. The first mode, labeled “affect heuristic I,” operates through a direct and immediate exposure to the affect triggering information without the assumption of any prior affective association by the subject (the first study by Finucane et al., 2000). The second mode, labeled “affect heuristic II,” delves into the impact of affect associated with past life experiences or the recently received information on the evaluation of costs and benefits (the second study by Finucane et al., 2000).

Green energy counteracts the greenhouse effect. However, the reluctance toward the green transformation could have a rational background in countries where energy from fossil fuels is more reliable than wind and solar power, especially the latter having a very poor capacity factor at this latitude. Additionally, people are still discovering the drawbacks of environmentally friendly energy sources, such as recycling used wind turbines (Stella, 2019) or the possible side effects of nighttime warming caused by heat redistribution by wind power (Miller and Keith, 2018). While climate change is a global problem, local concerns and idiosyncrasies should not be dismissed out of hand.

In the present research, we examine the perception of two kinds of risk: the environmental threats (ET) of hard coal mining and the personal safety hazards (PHs) of underground work.

The perception of the risk associated with ET (pollution, climate change, etc.) depends on the life experiences of respondents. People living in areas that are at low risk of pollution assess the environmental risk as smaller than those living in areas that are at high risk of pollution (Janmaimool and Watanabe, 2014). Similarly, dangerous work conditions increase the awareness of PHs (Goldberg et al., 1991). Salient, spectacular risks, such as airplane crashes or explosions, are better recognized than less tangible ones, such as the leakage of toxic substances (Cezar-Vaz et al., 2012). Affect seems to play an important role in risk assessments. Siegrist and Sütterlin (2014) noticed a divergent perception of natural vs. manmade hazards. Participants viewed 15 deaths due to the release of sulfur dioxide from a factory as a significantly more severe incident than 15 deaths due to the release of sulfur dioxide in a volcanic eruption. The same probability of work accidents (“*two fatalities per year on average*”) was deemed to be more acceptable for a photovoltaic power plant than for a similar-sized nuclear power plant, because of a much more positive affect evoked by solar technology compared to nuclear technology. Solar power is perceived as “*closer to nature compared with other technologies (e.g., nuclear power)*.”

Hinea et al. (2007) interviewed the residents of a small Australian city who experienced winter smoke pollution problems due to the extensive usage of wood heaters. Those residents who used wood heaters, when compared to the nonusers, perceived wood heating to pose fewer health risks. They were not supportive of the imposition of heavy fines on households that emitted excessive smoke. The research stressed the fact that the positive affect associated with an outdated technology could constitute a similar barrier to technological changes as the negative affect was associated with certain modern technologies.

Hard coal mining has played a prominent role in the Polish economy for centuries, and remains an important sector of the economy, providing social prestige and high earnings. Hard coal miners (CMs) enjoyed several economic and legal privileges, such as a profitable pension system and financial and nonfinancial occupational bonuses. On the other hand, the mining and usage of hard coal have been increasingly associated with a negative impact on the environment – both direct (caused by mining activity itself) and indirect (externalities caused by burning fossil fuels), and hard coal mining has always been perceived as a dangerous occupation connected with a heightened risk of work accidents, including deadly disasters.

In line with Hinea et al. (2007), we hypothesized that, due to the affect heuristic, those involved in the coal mining industry would automatically assess its benefits as high and its risk as low, whereas those not involved in the coal mine industry would assess its benefits as low and its risk as high.

Hard CMs who are working underground may be the beneficiaries of the hard coal industry but they also bear the high personal hazard of working in a mine. It should be underlined that people have a strong need for consistency within their own beliefs and behavior. If miners were critical of the hard coal industry, they might feel a dissonance between what they do and what they think. Inconsistency leads to disharmony which people strive to avoid or reduce. People are known to change their opinions, not their behavior (Festinger, 1962).

Thus, miners might assess the benefits of the hard coal industry as high but the personal and environmental risk as low due to the cognitive dissonance reduction, not the affect heuristic. However, reducing the dissonance requires a great deal of cognitive effort. It is a slow process. On the other hand, affect appears rapidly, unconsciously, and effortlessly. If people exhibit the affect heuristic, they do not need to reduce the cognitive dissonance. We intended to test whether the affect heuristic is strong enough to be responsible for a potential negative correlation between the perceived benefits and an assessment of the personal hazard of hard coal mining. We introduced three groups of respondents: hard CMs, fish processing plant workers, and hard coal mine security guards (SGs). The first group has benefited from the mining industry and is exposed to the PHs. The second group neither has benefited from the mining industry nor has it been exposed to the personal hazards. The third group has benefited from the mining industry but is not exposed to the hazards of underground work. If miners and mine SGs have the same assessment of PHs, it will mean that miners do not reduce cognitive dissonance.

While assessing the environmental impact of hard coal, we focused on its direct effects: the devastation of landscape and pollution. These negative externalities of the hard coal industry are widely recognized by the public in Poland. Therefore, the respondents’ opinions of their magnitude seem to be a reliable indicator in assessing the real cost of the hard coal industry. Surveying people on the indirect effects, such as climate change, would be much more challenging as the results might easily be distorted by political views and ideological inclinations (Duarte et al., 2015). The other part of the questionnaire is devoted to the perception of the contributions of the coal mining industry to the Polish economy in general. These seemed to be a sensitive topic as the glorious past of “black gold” (the metaphorical name for hard coal in Poland) clashes with the present grim economic and environmental realities. Finally, we examined the evaluation of hard coal mine PHs both in relative terms – that is, how dangerous mining is compared to other industries, and regarding the assessment of specific risk – that is, if methane explosions are a significant risk to miners. The PHs of hard coal mining are well-known to the general public, and mining disasters receive a broad media coverage.

We formed three hypotheses, Hypotheses 1 and 2 refer to the second study by Finucane et al. (2000), where participants were informed of the benefits (risks) of particular technologies and then asked to perform their own evaluation of the risks (benefits). A negative correlation occurred between the evaluated benefits and risks of a particular technology. The present approach is based on the past experience of respondents. Hypothesis 1 refers to emotions affecting judgments in a predictable way. Positive experience (i.e., a well-paid and prestigious occupation) of the hard coal industry impacts the positive overall evaluation of the area due to the associated positive affect.

*Hypothesis* 1: Due to the affect heuristic, beneficiaries of hard coal mining (miners and mine SGs) estimate the benefits to the economy (BE) from hard coal usage to be higher than non-beneficiaries [fish processing factory workers (FWs)].

*Hypothesis* 2: Due to the affect heuristic, beneficiaries of hard coal mining (miners and mine SGs) estimate the negative impact of hard coal mining on the environment to be smaller than people who have not benefited from the hard coal industry (fish processing FWs).

Consequently, in line with the affect heuristic, hard coal mining beneficiaries (miners and mine SGs) should estimate the personal hazard of underground work to be lower than people who have not benefited from coal mining (fish processing FWs).

If miners who are directly exposed to the personal hazard of underground work estimated the personal hazard to be lower than people who are not exposed to the hazard (SGs and fish processing FWs) do, which would mean that another effect plays a role: miners could reduce their cognitive dissonance. Distinguishing between cognitive dissonance and the affect heuristic is made possible by means of analyzing the responses of the SGs – respondents who are not exposed to personal hazard, and thus do not need to reduce any dissonance, but whose perception of risk may be distorted by the associated benefits. Thus, Hypothesis 3 has been added.

*Hypothesis* 3: Due to the affect heuristic, people who have benefited from the coal mining industry (both hard CMs and mine SGs) estimate the PH of underground work to be lower than people who have not benefited from the hard coal industry (fish processing FWs) do.

Confirmation of Hypothesis 3 would mean that both hard CMs and security staff exhibit the affect heuristic. A positive evaluation of hard coal diminishes the perceived PH. Hypothesis 3 assumes that the positive affect concerning the hard coal industry overrides not only common knowledge of the high rate of accidents in the mining industry but also personal experience of hazardous work.

The impact of the hard coal industry on the environment and economy is not measurable. In contrast, the PHs of underground work can be evaluated with objective measures, according to which mining and extraction remain the most dangerous occupational category in Poland. Based on this, we can safely assume that those who estimate the safety risks as low are definitely underestimating them.

## Materials and Methods

### Participants

The main analysis was to provide a comparison among the three groups. It was assumed that the smallest meaningful effect of the analysis was to detect at least 5% of the variance explained by the differences between the groups. This means that the partial *η*^2^ effect size measure was equal to 0.05. In order to calculate the required sample size, the WebPower library working in the R Statistics 4.0.3 software environment was used (Zhang and Yuan, 2018). The value of the partial *η*^2^ effect size measure was converted to Cohen’s *f* effect size measure, which was equal to 0.23. Assuming that statistical power seemed to be equal to 0.80 and the conventional cutoff point for statistical significance was equal to 0.05, the total sample size required for the ANOVA to be statistically significant was equal to at least 186 participants.

Research was conducted in two hard coal mines and in a fish processing plant. A total of 298 subjects participated in the study. The participants were from the three different groups: hard CMs (*n* = 99, all males), SGs from a hard coal mine (*n* = 98, 19 females), and workers from a fish processing plant (*n* = 101, 32 females). Since the questionnaire was personally administered to the available shift workers with cooperation from their employers, the response rate was 100%. The age profiles of our respondents are presented in Table 1.

**Table 1:** Age distribution of participants in the sample frequencies.

Age	Miners	Workers	Security guards
<25	0	6	1
25–30	30	27	15
31–55	34	34	29
>55	35	34	53

### Procedure and Materials

The survey included 18 questions, which were divided into three subscales. This division was made by a team of three experts who grouped the items forming the subscales. The first group of five questions measured the economic benefits of the hard coal industry, the second group of six questions measured perceived PHs in hard coal mining, and the third group of seven items compared the externalities and ET of the hard coal mining industry to those of other sources of pollution. We refer to the first scale as the perception of BE, to the second as the perception of PHs, and the third as the perception of ET. Participants gave their answers on an 11-point Likert scale ranging from −5 (completely disagree) to +5 (completely agree). Participants also had an option to answer “no opinion”; however, we noted only four such responses in the entire sample for all questions and decided to retain all participants in the sample. All questions are included in Supplementary Material.

The present study is close to a field study, so a convenient sample has been used. The sample is not representative. We did not influence the demographic composition of the sample, but – thanks to a high response rate – we avoided a selection bias.

## Results

The internal consistency of the three scales was very high as measured by the Cronbach’s alpha coefficient: BE scale alpha = 0.973 CI [0.968; 0.978], PH scale alpha = 0.952 CI [0.943; 0.960], and ET scale alpha = 0.959 CI [0.951; 0.966]. In none of the scales, would deleting any of the questions have yielded a higher alpha.

The mean scores from the three scales were highly correlated throughout the sample. The score on the scale measuring the BE correlated negatively with the perception of PHs of the hard coal industry (*r* = −0.818; *p* < 0.001), and negatively with the scale measuring ET (*r* = −0.889; *p* < 0.001) as well. The score on the PH scale correlated negatively with the score on the ET scale (*r* = −0.866; *p* < 0.001). The correlation pattern in the subsamples is presented in Table 2. Almost all correlations are highly significant, indicating a negative relationship between benefits and ET and a positive relationship between ET and personal hazards. Also, there is a general negative correlation between the perception of personal hazards and BE, although it is insignificant for hard CMs. Furthermore, we compared the correlation coefficients from the different groups to test whether they differ in terms of their strength. The correlation between the perception of personal hazards and BE is significantly stronger in the group of SGs when compared to this relationship within the group of FWs (*Z* = 4.212 *p* < 0.001). Comparing the same relationship within the group of hard CMs and SGs, the difference between the relative strengths is not significant (*Z* = 1.414; *p* = 0.079).

**Table 2:** Correlations among the three measures in a sample across the three groups.

	Coal miners		Fish factory workers		Security guards	
	Personal hazard	Environmental threats	Personal hazard	Environmental threats	Personal hazard	Environmental threats
Benefits to the economy	−0.019	−0.441^**^	−0.680^**^	−0.565^**^	−0.219^*^	−0.455^**^
Personal hazard		0.479^**^		0.668^**^		0.459^**^

* *p* < 0.05;

** *p* < 0.001.

**Figure 1:**
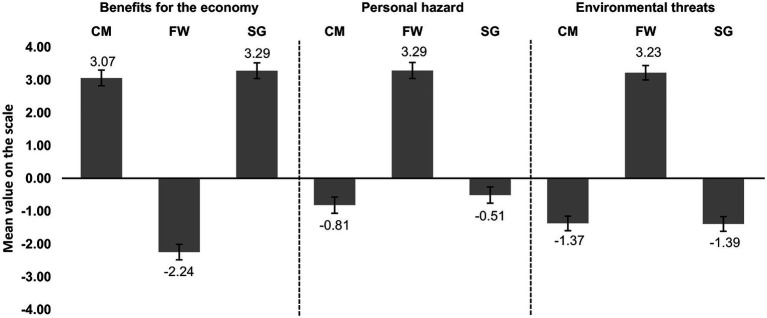
Means with 95% CIs across the three dependent variables [the perception of benefits to the economy (BE), personal hazard (PH), and environmental threats (ET)] in groups of hard coal miners (CMs), fish processing factory workers (FWs), and security guards (SGs).

Finally, we compared the mean scores of the scales across the three groups using 3 × 3 (three dependent variables, i.e., the scores from the BE, PH, and ET scales, in three groups, i.e., miners, SGs, and FWs). All multiple comparisons were done by using Sidak’s correction. The overall multivariate ANOVA (MANOVA) score showed significant differences in mean scores across the three dependent variables in compared groups [*F*(6;588) = 77,329; *p* < 0.001; *η*^2^_p_ = 0.441; Pillai’s trace = 0.882]. We found significant differences among the three groups in scores for the BE scale [*F*(2;295) = 681,115; *p* < 0.001; *η*^2^_p_ = 0.822] and for the personal hazards scale [*F*(2;295) = 336,053; *p* < 0.001; *η*^2^_p_ = 0.695]. There were also differences in the perception of ET linked to the hard coal mining industry [ET scale, *F*(2;295) = 565,076; *p* < 0.001; *η*^2^_p_ = 0.793]. In all three dependent variables, the pattern of differences among the three groups is the same, i.e., there are no significant differences between the scores of hard CMs and mine SGs (BE, *p* = 0.161; PH, *p* = 0.279; ET, *p* = 0.995), and all other differences are significant at the level *p* < 0.001. The means of three scales across the three groups are presented in Figure 1.

Both hard CMs and mine SGs assessed the economic benefits of hard coal usage as high, the individual hazards of working in the mine as slightly lower than in other industries, and the detrimental impact of the mining industry on the environment as rather small. The evaluations by the fish processing plant workers revealed the opposite perspective: they consider mining to be costly to the state economy, and hazardous to both the environment and to individuals who are working underground. Across all the scales, no significant differences were apparent between hard CMs and mine SGs.

The first hypothesis assumed that both miners and mine SGs perceive the economic benefits of the mining industry as higher than fish processing plant workers. The hypothesis was confirmed. Not only there was a huge difference in the perception of economic benefits between mine workers (both miners and SGs) and fish processing plant workers but also the estimates were positive and negative for miners and fish processing plant workers, respectively.

The second hypothesis assumed that mine workers perceive a detrimental environmental impact of the mining industry as lower than fish processing plant workers. The hypothesis was confirmed. Not only there was a huge difference in the perception of environmental hazards between mine workers and fish processing plant workers, but also the estimates by mine workers were positive as opposed to negative in the case of fish processing plant workers. Interestingly, despite the fact that the hard coal mining industry is commonly considered to be environmentally harmful, mine workers perceived the ET arising from hard coal mining as being smaller than those of other industries.

The third hypothesis assumed that people who have benefited from working in the mining industry (CMs and mine SGs) would estimate the PHs in mining as lower than people who have not benefited from the mining industry (fish processing FWs). This has been confirmed in the present research. Both miners and mine SGs evaluated the PH as lower when compared to fish processing FWs. Personal hazard estimates made by the participants from the group of mine SGs were broadly similar to those of miners. These results offer support for the affect heuristic and additionally allow for the exclusion of alternative explanations, including a cognitive dissonance reduction, as mine SGs would not suffer from any dissonance in this regard. These results show that the affect heuristic is responsible for hard coal workers estimating low PHs.

## Discussion

Three groups of respondents were examined: hard CMs, fish processing FWs, and hard coal mine SGs. Hard CMs have benefited from the mining industry and are exposed to PH. Fish processing plant workers neither have benefited from the mining industry nor are exposed to personal hazard. Hard coal mine SGs have benefited from the mining industry but are not exposed to the hazards of underground work. We observed that the beneficiaries of hard coal mining (miners and mine SGs) estimated the benefits of hard coal usage to the economy to be higher than the non-beneficiaries (fish processing FWs) do. Hard coal mining beneficiaries (miners and mine SGs) estimated the negative impact of hard coal on the environment to be smaller than people who have not benefited from hard coal mining (fish processing FWs) do. What is more, respondents who have benefited from the coal mining industry (both SGs and hard CMs) estimated the PH of underground work to be lower than people who have not benefited from coal mining (fish processing FWs).

There were no significant differences between the responses of hard CMs and mine SGs. Both hard CMs and mine SGs perceived the economic benefits of hard coal as positive, the individual hazards of working in the mine as lower than in other industries, and the threats to the environment as minor. The evaluations by fish processing plant workers revealed the opposite. The above observations are a field application of previous research (Finucane et al., 2000). By predicting the respondents’ perception of the benefits of a technology (or industry), we were able to predict their opinion of the riskiness of that technology (or industry). Since miners and SGs are employed in the mining industry and are the recipients of appropriate remuneration, they obviously tend to perceive the benefits of hard coal to the economy as larger than the representatives of other industries do.

Interestingly, we observed that miners and mine SGs perceived both the risks of coal mining to the environment and PHs to be smaller than the representatives of any other industry, namely fish processing FWs. While the environmental risk assessment may seem to be subjective, the PH is more measurable. Both SGs and hard CMs estimated the PH of underground work to be lower than fish processing FWs did, with no significant difference between the scores of hard CMs and mine SGs. One may suspect that there are fish processing FWs (being unfamiliar with the realities of working in a coal mine) who wrongly estimate the risk associated with a given activity while miners and SGs have an accurate perception of the dangers they face every day. However, this is not borne out by reality. According to the Polish Central Statistical Office (GUS, 2019), “mining and extraction” remains as one of the most dangerous occupational categories, with twice the rate of work accidents than the construction industry and 10 times the rate of accidents than in services. Polish mines extract hard coal from relatively deep layers (on average 700 m/2200 ft below the surface) where the probability of tremors and methane explosions is high. An individual faced with cognitive dissonance will experience discomfort and may then engage in motivated cognitive processing to reduce the dissonance (for a review, see Hinojosa et al., 2016). Miners who, on the one hand, face the discrepancy between a stable job and high levels of personal hazard, and, on the other hand, could feel this type of dissonance. However, the socioeconomic reality in which hard coal industry workers live seems to be incompatible with the settings that trigger dissonance. Typical conditions that trigger cognitive dissonance involve a free choice and post-choice rationalization, especially when the choice is costly and there are many viable alternatives – such as buying an expensive, exotic car and later realizing that it does not live up to the hype. Hard coal mining is a hereditary occupation in Poland, with a high percentage of miners having relatives who also work in the mines. Some miners can trace the mining tradition in their families back for five generations. Hard coal mining in Silesia is considered a prestigious job, and miners rank just below physicians, university professors, and police officers in terms of prestige. Hard coal mines are also strategically important employers, patrons of some local social initiatives, and an important source of municipal tax revenues. Miners are proud of their work. Taking all this into account, it is rather unexpected that the work environment could be a source of cognitive dissonance in Silesian miners. Quitting the mining sector, for example, *via* restructuring and golden-handshake programs, sometimes results in professional inactivity rather than finding a different job (Szpor, 2017). When one makes a default and socially expected choice, the possibility that they will exhibit a cognitive dissonance is largely diminished. In reference to the present research, it is possible to say that miners could reduce their cognitive dissonance if they would estimate personal hazard to be smaller compared to mine SGs. After all, these are the miners who are exposed to PHs and not to the mine SGs. People reduce cognitive dissonance when they have skin in the game (Taleb, 2020). In other words, since mine SGs are not exposed to personal hazard, they do not need to reduce cognitive dissonance. SGs have no reason to rationalize the dangers of underground mining as they are not personally exposed to these risks. They neither face a moral dilemma nor need post-choice rationalizations. The verdict that miners who did not reduce cognitive dissonance is drawn indirectly and needs further empirical examination.

Seabright (2010) observed that the temporal and psychosocial distance of global warming draws too little moral concern. He has proposed to stress the short-term and personal consequences of global warming, thus eliciting a negative affect and overcome inaction. However, neither environmental impact nor personal hazard is “temporary or spatially” distant to hard CMs. Most of them live a very short distance from the workplace and might contemplate the environmental consequences of hard coal mining. They also might have experienced a work accident firsthand. The affect heuristic, being a powerful cognitive effect, makes anyone who sees the benefits of any technology, automatically minimize its risk. A very recent example of people who, while perceiving the riskiness of technology as high, automatically perceive its profitability as low comes from Deutsche Welle: “Anyone who has visited the site of the Chernobyl nuclear power plant and experienced the oppressive silence in the death zone around the damaged reactor and in the nearby city of Pripyat in northern Ukraine, once home to 50,000 people, might draw the conclusion that Merkel's decision 10 years ago was right” (Thurau, 2021). The affect heuristic, associated with the impact of past life experiences on the evaluation of costs and benefits, could be alternatively phrased as follows: individuals automatically ignore the less salient side of the story. Thus, the dangers of visibly beneficial activity lie within the blind spot of the individual whereas the more salient features of an activity (the low price of hard coal and its economic benefits to the local community) significantly distort the less tangible disadvantages (ET).

We should strive toward the modest goal of being able to identify and debrief the affect heuristic in its most prominent manifestations in political, economic, and social life, but we should not hope that anyone will ever be immune to this judgmental bias.

While the two papers that explore the concept of the affect heuristic have been cited more than 1,000 times each (Finucane et al., 2000; Slovic et al., 2002), this cognitive distortion remains almost unexplored in reference to environmental policies. Nevertheless, one cannot simply counter the favorable perception of the fossil fuel economy by providing vivid images of its dire impact on the environment and contribution to global warming. The affect heuristic works quickly and automatically. Thus, where the salient benefits are at play, the most efficient way to make people accept the transformation of the energy sector is to assure them that they will not be worse off. This is called a Kaldor-Hicks improvement. What is more, “if workers derive satisfaction from their particular kind of work, and are obliged to change their employment, something more than their previous level of income will be necessary to secure their previous level of enjoyment (…)” (Kaldor, 1939, p. 551).

## Data Availability Statement

The raw data supporting the conclusions of this article will be made available by the authors, without undue reservation.

## Ethics Statement

The studies involving human participants were reviewed and approved by Human Research Ethics Committee (HREC) at Kozminski University. Written informed consent for participation was not required for this study in accordance with the national legislation and the institutional requirements.

## Author Contributions

All authors contributed to the present work. The authors took part in drafting or revising it critically for important intellectual content and approved the final version to be published. Furthermore, all authors ensured that the questions related to the accuracy or integrity of any part of the work were appropriately investigated and resolved. PZ: substantial contribution to the conception and design of the research and the interpretation of data. WB: data analysis and visualization. BD: interpretation of data. KW: data acquisition.

### Conflict of Interest

The authors declare that the research was conducted in the absence of any commercial or financial relationships that could be construed as a potential conflict of interest.

## References

[ref1] Cezar-Vaz M. R. Pereira Rocha L. Alves Bonow C. Santos Da Silva M. R. Cezar Vaz J. Silveira Cardoso L. (2012). Risk perception and occupational accidents: a study of gas station workers in Southern Brazil. Int. J. Environ. Res. Public Health 9, 2362–2377. 10.3390/ijerph9072362, PMID: 22851948 PMC3407909

[ref2] Duarte J. L. Crawford J. T. Stern C. Haidt J. Jussim L. Tetlock P. (2015). Political diversity will improve social psychological science. Behav. Brain Sci. 38, 1–58. 10.1017/S0140525X14000430 25036715

[ref3] Festinger L. (1962). Cognitive dissonance. Sci. Am. 207, 93–106. 10.1038/scientificamerican1062-93 13892642

[ref4] Finucane M. L. Alhakami A. Slovic P. Johnson S. M. (2000). The affect heuristic in judgment of risks and benefits. J. Behav. Decis. Mak. 13, 1–17. 10.1002/(SICI)1099-0771(200001/03)13:1<1::AID-BDM333>3.0.CO;2-S

[ref5] Goldberg A. I. Dar-El E. M. Rubin A. E. (1991). Threat perception and the readiness to participate in safety programs. J. Organ. Behav. 12, 109–122. 10.1002/job.4030120204

[ref6] GUS (2019). Wypadki przy pracy w 2018 roku [eng. Occupational Accidents in 2018]. Available at: https://stat.gov.pl/obszary-tematyczne/rynek-pracy/warunki-pracy-wypadki-przy-pracy/wypadki-przy-pracy-w-2018-roku,3,34.html (Accessed June 9, 2021).

[ref7] Hinea D. W. Marks A. G. D. Nachreiner M. Gifford R. Heath Y. (2007). Keeping the home fires burning: the affect heuristic and wood smoke pollution. J. Environ. Psychol. 27, 26–32. 10.1016/j.jenvp.2007.01.001

[ref8] Hinojosa A. S. Gardner W. L. Walker H. J. Cogliser C. Gullifor D. (2016). A review of cognitive dissonance theory in management research: opportunities for further development. J. Manag. 43, 170–199. 10.1177/0149206316668236

[ref9] Hsee C. K. (1998). Less is better; when low-value options are valued more highly than high-value options. J. Behav. Decis. Mak. 11, 107–121.

[ref10] Janmaimool P. Watanabe T. (2014). Evaluating determinants of environmental risk perception for risk management in contaminated sites. Int. J. Environ. Res. Public Health 11, 6291–6313. 10.3390/ijerph110606291, PMID: 24937530 PMC4078580

[ref11] Kahneman D. (2011). Thinking Fast and Slow. New York: Farrar, Straus and Giroux.

[ref12] Kaldor N. (1939). Welfare prepositions of economics and interpersonal comparisons of utility. Econ. J. 49, 549–552. 10.2307/2224835

[ref13] Miller L. M. Keith D. W. (2018). Climatic impacts of wind power. Joule 2, 2618–2632. 10.1016/j.joule.2018.09.009

[ref14] Seabright M. A. (2010). The role of the affect heuristic in moral reactions to climate change. J. Global Ethics 6, 5–15. 10.1080/17449621003701410

[ref15] Shefrin H. (2005). A Behavioral Approach to Asset Pricing. Amsterdam: Elsevier Academic Press.

[ref16] Siegrist M. Sütterlin B. (2014). Human and nature-caused hazards: the affect heuristic causes biased decisions. Risk Anal. 34, 1482–1494. 10.1111/risa.12179, PMID: 24576178

[ref17] Slovic P. Finucane M. Peters E. MacGregor D. G. (2002). “The affect heuristic,” in Heuristics and Biases. The Psychology of Intuitive Judgements. eds. Gilovich T. Griffin D. Kahneman D. (Cambridge: Cambridge University Press), 397–420.

[ref18] Stella C. (2019). Unfurling the Waste Problem Caused By Wind Energy. NPR. Available at: https://www.npr.org/2019/09/10/759376113/unfurling-the-waste-problem-caused-by-wind-energy (Accessed June 9, 2021).

[ref19] Szpor A. (2017). Coal Transition in Poland. An historical case study for the project “Coal Transitions: Research and Dialogue on the Future of Coal”. IDDRI and Climate Strategies, Institute for Structural Research.

[ref20] Taleb N. N. (2020). Skin in the game: Hidden asymmetries in daily life. Random House Trade Paperbacks.

[ref21] Thurau J. (2021), Opinion: After Fukushima, Germany must avoid nuclear energy, Deutsche Welle. Available at: https://www.dw.com/en/opinion-after-fukushima-germany-must-avoid-nuclear-energy/a-56828921 (Accessed June 9, 2021).

[ref22] Zhang Z. Yuan K.-H. (2018). Practical Statistical Power Analysis using WebPower and R. Granger, IN: ISDSA PRESS.

