# Supplementary material for: How Miners and Other Professional Groups Perceive the Benefits and Risks of Hard Coal Mining: A Study on the Role of the Affect Heuristic
Source: Front Psychol. 2021 Jun 29;12:656960. doi: 10.3389/fpsyg.2021.656960 (PMC8276241; doi:10.3389/fpsyg.2021.656960)
Supplement: Supplementary file 1 [file Data_Sheet_1.pdf]

Supplementary Material

Questionnaire on the Coal Mining Industry

Dear Sir/Madam,

Please fill out the following questionnaire on the coal mining industry. Your answers are anonymous and will be used solely for scientific purposes.

The questionnaire should be completed as follows: please read the question and then mark your answer on the scale below, where:

the answer '–5' means "I strongly disagree"

the answer '+5' means "I strongly agree"

You may also select the option 'I have no opinion.'

[sample question format]

Poland is developing thanks to the coal mining industry.

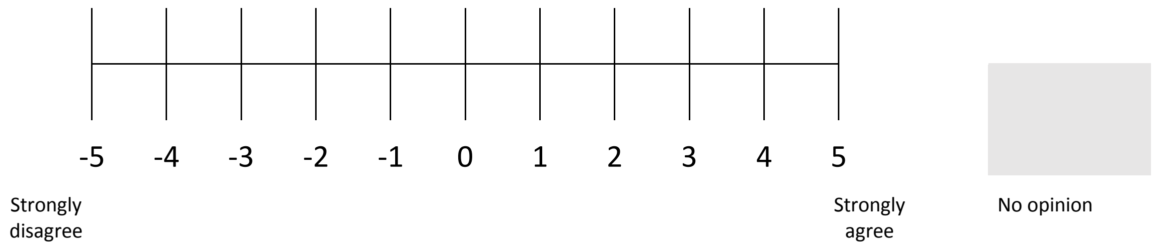

I. Questions about the economic benefits of hard coal

1. Poland is developing thanks to the coal mining industry.
2. Hard coal mines play an important role in the energy sector.
3. Hard coal is a good source of energy.
4. Hard coal mines play a significant role as an employer in southern Poland.
5. The use of hard coal as an energy source is beneficial to the economy.

II. Questions about the personal safety hazard of working underground

6. In mining, people are more often exposed to health hazards at work than in other jobs.
7. In mining, people are exposed to a greater number of accidents at work than in other jobs.
8. Miners are more likely to suffer injuries than other workers.
9. The hard working conditions in the mine may cause permanent health damage to miners.
10. Tremors that occur in mines threaten the lives of miners.
11. Miners are at risk of losing their lives due to possible coal dust explosions.

III. The impact of the hard coal mining on the environment

12. Coal mines are much more damaging to the environment than other industrial plants.
13. Hard coal waste released during the operation of power plants is more harmful than waste generated by nuclear power plants.
14. Seismic activity due to the extraction of hard coal is very harmful to the environment.
15. Extracting hard coal can adversely affect buildings that are located above mines.
16. Mines have a negative impact on the climate of Upper Silesia.
17. Mines have a much greater impact on river wastewater pollution than other industrial plants.
18. Mines have a negative impact on the yield of agricultural land.
